# Information Needs at the Beginning of Foraging: Grass-Cutting Ants Trade Off Load Size for a Faster Return to the Nest

**DOI:** 10.1371/journal.pone.0017667

**Published:** 2011-03-09

**Authors:** Martin Bollazzi, Flavio Roces

**Affiliations:** 1 Department of Behavioral Physiology and Sociobiology, Biocenter, University of Würzburg, Würzburg, Germany; 2 Department of Entomology, Faculty of Agronomy, Universidad de la República, Montevideo, Uruguay; 3 Centro Universitario de Rivera, Universidad de la República, Rivera, Uruguay; University of Alabama, United States of America

## Abstract

**Background:**

Acquisition of information about food sources is essential for animals that forage collectively like social insects. Foragers deliver two commodities to the nest, food and information, and they may favor the delivery of one at the expenses of the other. We predict that information needs should be particularly high at the beginning of foraging: the decision to return faster to the nest will motivate a grass-cutting ant worker to reduce its loading time, and so to leave the source with a partial load.

**Principal Findings:**

Field results showed that at the initial foraging phase, most grass-cutting ant foragers (*Acromyrmex heyeri*) returned unladen to the nest, and experienced head-on encounters with outgoing workers. Ant encounters were not simply collisions in a probabilistic sense: outgoing workers contacted in average 70% of the returning foragers at the initial foraging phase, and only 20% at the established phase. At the initial foraging phase, workers cut fragments that were shorter, narrower, lighter and tenderer than those harvested at the established one. Foragers walked at the initial phase significantly faster than expected for the observed temperatures, yet not at the established phase. Moreover, when controlling for differences in the fragment-size carried, workers still walked faster at the initial phase. Despite the higher speed, their individual transport rate of vegetable tissue was lower than that of similarly-sized workers foraging later at the same patch.

**Conclusions/Significance:**

At the initial foraging phase, workers compromised their individual transport rates of material in order to return faster to the colony. We suggest that the observed flexible cutting rules and the selection of partial loads at the beginning of foraging are driven by the need of information transfer, crucial for the establishment and maintenance of a foraging process to monopolize a discovered resource.

## Introduction

Collective foraging by social insects is a complex process that involves assessment of resource quality by workers, decision making, food carriage and information transfer (e.g., bees [1], [2]; bumblebees [3]; wasps [4]; ants [5], [6]). A social insect colony does not “decide” about, for instance, the selection of a given food source, yet the colony's foraging responses arise from the decisions made by each individual worker, and from the interactions among colony members.

Central for the understanding of social foraging is the fact that most of the resources collected by foragers are not for their own consumption, but fed to the brood and to nestmates, or stored. Whatever the final end of the collected resources, individual foragers are expected to behave in a way that maximizes food intake at the colony level, because one can assume that the foraging performance of a colony, measured as the delivery rate of food, correlates with colony fitness. The analysis of individual foraging performance, and its extrapolation to account for overall colony responses, is nonetheless not straightforward because returning foragers deliver two commodities to the colony: food and information [7]–[9]. Because food gathering consumes both time and energy, one could a priori expect a trade-off between time spent collecting on one side, and time used for information transfer on the other side, if colony foraging performance is enhanced through communication, even at the expense of a reduced individual foraging performance. In this scenario, workers could potentially favor the delivery of one of these commodities at the expenses of the other, i.e., workers may spend less time collecting at a food source, thus reducing their own food-delivery rate, but allocate more effort to pass information about the discovery, thus leading to a higher food intake at the colony level. An early return to the nest shortens the foragers' time to transmit information both via recruitment signals and through direct interactions with nestmates, and may allow potential recruits to get quickly informed about a food discovery [9]–[11]. When this dual aspect is kept in mind, it becomes clear that the optimal policies for delivering each of these two commodities, food and information, might be different, and that the maximization of food provisioning at the individual level, with the associated collecting and carrying costs, is incompatible with a rapid delivery of information [9], [12], [13]. The extreme strategy of time saving at the food source for information transfer would be to return completely unladen to the nest, but displaying recruitment behavior, as described for leaf-cutting ants [14].

Leaf-cutting ants (Attini, genera *Atta* and *Acromyrmex*) have repeatedly been used as a model system to explore how communication demands influence foraging decisions, because workers can make, through flexibility in cutting behavior, their own decisions about the fragment size to be cut [15]. Leaf-cutting ants are conspicuous herbivores of the Neotropics that cut vegetation into small fragments, which are then transported to the nest as substrate for a symbiotic fungus. It has repeatedly been observed that polymorphic leaf-cutting ants, because of their geometric mode of cutting roughly semicircular fragments, frequently cut leaf pieces that correspond in size to that of the ants' body [16]. However, not all workers cut fragments of maximal size, indicating the involvement of more flexible mechanisms of load-size determination. For instance, foragers cut smaller fragments from sources located close to the nest [17], and also from hard leaves [18]–[21]. In addition, leaf-cutting ant workers were shown to use flexible cutting rules at a newly-discovered, highly-attractive food source: workers shorten their foraging time by cutting smaller fragments and return to the nest with partial loads, intensively recruiting additional nestmates, as discussed in the framework of the so-called information-transfer hypothesis [2], [18]. This hypothesis states that at newly-discovered food sources, foragers select loads that do not maximize their individual intake rates, but allow them to return earlier to the colony for information transfer. Foragers' performance as food carriers is therefore reduced, but the colony as a whole increases its harvesting rate due to the workers that gained information and participate in the resource-gathering activity [2], [9], [15], [18], [22]. Thus, saving cutting time/energy because of the selection of small leaf fragments contributes to speed-up information transfer.

So far, the extent to which the decision to transfer information about a food discovery influences individual cutting behavior was only analyzed under controlled laboratory conditions, and only for leaf-cutting ants (reviewed in [22]). Upon discovery of a highly-attractive food source, they are known to cut smaller leaf fragments and to engage in intense recruitment behavior, which leads to a faster build-up of workers at the source [9], [18], [22]. While variation in leaf fragment size is necessarily associated with different cutting effort in leaf-cutting ants that cut semicircular fragments, the situation is rather different for grass-cutting ants. Workers of grass-cutting ants climb on a grass blade and cut across its width, which results in the selection of a longish, more or less rectangular grass fragment. Therefore, cutting length is represented by the grass width, which usually does not differ very much along the blade except at its tip. Hence, cutting a larger (longer) fragment does neither imply a higher cutting effort nor a longer cutting time, if grass toughness remains unchanged along the blade. Grass-cutting ant workers may therefore harvest more material per unit cutting effort by simply cutting longer fragments.

Grass-cutting ants offer a unique opportunity to investigate whether workers use flexible cutting rules and compromise their load delivery for a faster return to the nest, because changes in load-size determination (fragment length) are not associated with additional costs or energy savings, as outlined above [23]. In addition, since workers do not anchor their hind legs at the grass end while cutting, body size poses no upper limit to the fragment size to be cut, as it is the case for leaf-cutting ants. Differences in fragment length are known to affect walking speed and individual delivery rates [24], [25], so that the selected fragment size is expected to largely influence the duration of a worker's foraging cycle.

When should information be worth transferring? We predict that the needs for information about a food discovery should be particularly high at the beginning of foraging, in order to allow a collective foraging process to get established. This study explores the extent to which grass-cutting ant workers use different cutting rules depending on the foraging phase, and save time as a response to the high demands for information transfer at the beginning of each daily foraging process. For that, we first quantified the foraging dynamics of several field colonies of the grass-cutting ant *Acromyrmex (Moellerius) heyeri*, counting the number of outgoing, returning laden and unladen workers, as well as the rates of their head-on encounters, at two distinct phases: initial and established foraging. These two phases are expected to be associated with different demands for information transfer: high at the beginning of a foraging process, and low on well-established trails [9], [11], [14], [26], [27]. In addition, the sizes of the grass fragments cut by workers at the two different foraging phases were recorded, as well as the workers' walking speed, allowing the later calculation of the individual gross transport rates of vegetable tissue. To control for the marked differences in environmental temperature during the field measurements, which are expected to largely influence walking speed, the relationship between walking speed and temperature was established over a wide range of temperatures in a laboratory colony, thus allowing the calculation of Q_10_-values for walking speeds, and the standardization of carrying performance at a given temperature for meaningful comparisons. Finally, both handling and cutting times by workers offered similar grasses as those harvested in the field were measured in a laboratory colony, to have a more comprehensive picture of the total time investment of single workers at both the initial and established foraging phases.

## Methods

The field study to quantify foraging dynamics and fragment-size determination was conducted between March and November 2009 in Tabaré, Florida, Uruguay (33°21′33.74″S, 55°35′33.38″W). A total of 15 *Acromyrmex heyeri* colonies were investigated. Counts of outgoing, returning (laden), returning (unladen) workers were performed, as well as records of walking speed, head-on encounters, fragment size and body size of laden workers. Two different phases along a natural daily foraging process were first determined. The “initial foraging phase” was considered to begin as soon as the first laden worker was observed to walk to the nest coming from the foraging area. The “established foraging phase” was considered to begin 2.5 hours later, based on the counts as presented in the results. Three to four hours later, foraging activity ceased due to the high environmental temperature, and not because of exhaustion of the available grasses. Data were collected on each colony's main foraging trail, 2 m away from the nest.

### Traffic flow and head-on encounters at the two foraging phases

Traffic flows of outgoing and returning (both unladen and laden) workers were determined along the colony's main foraging trail at regular time intervals, as well as the number of head-on encounters between focal workers and nestmates coming from the opposite direction, for both the initial and established foraging phases. Counts were performed on 12 *A. heyeri* field colonies during November 2009. At the measurement point, workers passing by were counted for 5 minutes, beginning at the time in which the first laden worker was observed to walk to the nest (initial phase). A second count was performed in the same colony at the established foraging phase. A similar procedure was used for the remaining 11 colonies. Immediately after each of the two counts, 12 randomly-selected workers (4 outgoing, 4 laden and 4 unladen walking to the nest) were carefully observed as they walked along a 30 cm trail section, and both the number of contacts (head-on encounters) with workers coming from the opposite direction, and the time elapsed to walk along the trail section, were recorded, so as to calculate the contact rates. Since the probability of contacting a worker directly depends on the number of workers coming from the opposite direction, and this number is expected to depend on the foraging phase considered, comparative values were obtained by dividing the contact rates (contacts*s^−1^) by the traffic flow (number of workers*s^−1^), which was recorded as indicated above.

In order to quantify more accurately the traffic dynamics as a function of day time, traffic flows were measured in an additional colony every 20 min throughout both phases, over six consecutive days.

### Fragment-size determination, walking speed and carrying performance at different temperatures

Walking speed of 10 laden workers was measured over 30 cm on a smooth trail section, once for each of the 15 colonies investigated during March 2009, for both the initial and established foraging phase. Immediately thereafter, laden ants were collected, placed singly in 10 ml vials, and frozen. The scored ants were randomly chosen by collecting every fifth forager that passed by the collecting point. Considering that the collection of 10 ants took approximately 15 min in each foraging phase, no more than two colonies could be investigated each day, so as to keep the measurements at both the initial and established foraging phase within a time window of 30 min. Only workers carrying fresh grass fragments, i.e., those cut immediately before, were considered for further analysis. Workers carrying falling flowers or dry grasses were ignored because these items were directly collected without the involvement of cutting. Workers and their loads were later weighed to the nearest 0.1 mg. Both fragment length and width were measured with a field binocular to the nearest 0.01 mm. For each collected ant, day time and ambient temperature (0.01°C resolution) at the ground level beside the collecting point were precisely determined via the use of temperature Dataloggers (Tinytag, Gemini Data Loggers). The obtained data allowed the calculation of the individual transport rates (mass of vegetable tissue carried per mm and per second) at the actual foraging temperatures.

### Comparing carrying performance at the two foraging phases: standardization of walking speeds at 15°C

Marked differences in environmental temperature during the field measurements, which are expected to largely influence walking speed, were taken into account by analyzing the relationship between temperature and walking speed over a wide range of temperatures, in workers from a laboratory colony. These data allowed the calculation of Q_10_-values for walking speed, for temperatures ranging from 15 to 35°C, at which foraging activity actually occurred in the field. Field values of speed could therefore be transformed at a given temperature, allowing the comparison of transport rates between the foraging phases irrespective of temperature. During May–June 2009, the relationship between speed and temperature was established by recording walking speeds of laden workers, in independent assays, at 15°C, 20°C, 25°C, 30°C and 35°C. For that, an *A. heyeri* colony was collected at the same location where the field experiments were performed, transported to the Department of Behavioral Physiology and Sociobiology at the University of Würzburg, Germany, and maintained in a climatic room under 25°C and 12∶12 Light∶Dark cycle. In the laboratory, the colony was allowed to collect grasses, and after a foraging column was well established, the time spent by 50 laden workers as they walked across a 50 cm section of the 2 m long wooden bridge connecting the colony with the foraging arena was recorded, for each of the temperatures assayed. To control for the effects of the load size on speed, standardized, previously-cut fragments of the grass *Festuca rubra* (Poaceae) in the size range harvested by field colonies (9.7±1.7 mm long; 0.61±0.30 mm wide; mean±SD) were offered on the foraging arena.

The dependence of walking speed on temperature was expressed by calculating the Q_10_-values for a given temperature interval, with the equation: Q_10_ = 10*exp[10*(logV_t2_−logV_t1_)/(T_2_−T_1_)], where V_t1_ and V_t2_ are the speeds at the temperatures T_1_ and T_2_, respectively. The Q_10_-values were then used to convert the walking speeds measured in the field at different temperatures during both foraging phases, to comparable values at 15°C, as follows: V_15_ = 10*exp[logQ_10_*(15−T_1_)/10+logV_t1_], where V_t1_ is the walking speed at temperature T_1_ and the Q_10_-value is that including the temperature interval 15−T_1_.

### Time investment before load carriage: Cutting and lift-up times

Time investment during foraging does not only include the travel time, as indicated above, but also cutting and handling (lift-up) times. Although no direct measurements of the time spent by workers during cutting could be done in the field, grass toughness, measured as tissue area density (fragment mass divided by fragment area, in mg*mm^−2^), is expected to largely determine the average cutting time by workers [18]. Based on the tissue area density measured for the fragments collected in the field, grasses with a similar area density were offered to workers from the laboratory colony, and the time needed to cut one millimeter of tissue was recorded. Such measurements allowed the cutting time workers spent under field conditions to be indirectly, but properly estimated. In order to account for the effect of the different ambient temperatures recorded at the two foraging phases, cutting times were recorded at 15°C and 25°C in independent laboratory series.

Handling (lift-up) times, i.e., how long a worker needs to bring the harvested fragment into an upright position, is expected to largely depend on fragment size, because of the torque that the worker must overcome during the collection. To investigate whether fragment size affects lift-up times, paper fragments with an average length, width and mass similar to the fragments harvested in the field were offered to workers from the laboratory colony during a foraging cycle. The time needed by foragers to lift-up the fragments from the floor to the upright position was recorded for fragment sizes that matched those of both the initial and established foraging phases. In order to stimulate workers to readily pick-up the paper fragments, they were previously impregnated with diluted orange juice (30% in water) and dried. As for cutting times, lift-up times were recorded at 15°C and 25°C in independent laboratory series.

## Results

### Traffic flow and head-on encounters at the two foraging phases

**Figure 1:**
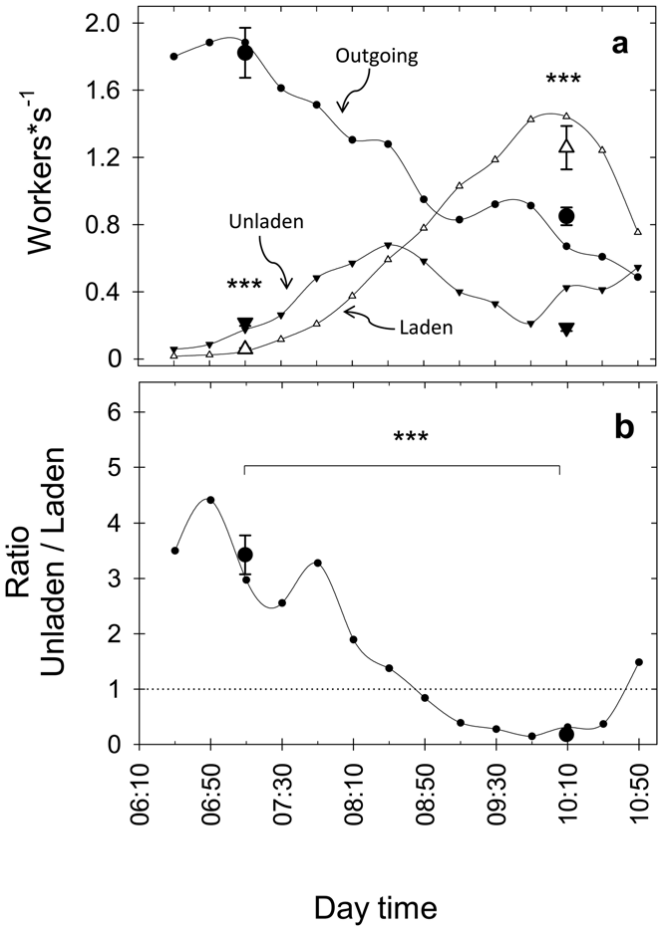
Traffic flows of outgoing, returning laden and unladen workers in field colonies. **A**) *Lines*: average traffic flow recorded every 20 minutes in a single colony during six consecutive days. *Large symbols*: average number of workers (mean±SD) from single measurements on 12 colonies at both the initial (07:10h) and established (10:10h) foraging phases. **B**) Ratio between unladen and laden returning workers. *Line*: average ratio obtained from the counts every 20 minutes in a single colony, during six consecutive days. *Large black circles*: average ratio from single measurements on 12 colonies at both the initial (07:10h) and established (10:10h) foraging phases. Asterisks indicate a statistical difference at p<0.001.

At the beginning of the counts at sunrise, several outgoing *A. heyeri* workers were already observed on the trail. The first returning laden workers arrived at the counting point usually around 6:30AM. The number of returning workers (both laden and unladen) increased, and the number of outgoing workers decreased with day time, and the morning foraging activity ended approximately 5 hours later, around 11:30 AM. The regular counts of traffic flows on a single colony allowed the clear-cut differentiation of two foraging phases: the initial one, before 8:30 AM, in which the number of outgoing workers still surpassed that of returning laden or unladen workers, and the established foraging phase, later than 8:30 AM, in which outgoing workers decreased in number and were surpassed by the sum of laden plus unladen workers (Fig. 1A, lines).

During the initial foraging phase, workers were likely to return to the nest without transporting a load (Fig. 1A). The average number of returning unladen workers was higher than the number of laden workers during the initial phase, yet the opposite occurred at the established phase. Such a pattern was also observed in the 12 additional field colonies in which single counts at the two foraging phases were performed (Fig. 1A, triangles, unladen vs. laden; initial: paired t-test, t_11_ = 9.53, p<0.001; established: paired t- test, t_11_ = 8.25, p<0.001). The ratio between unladen and laden workers was higher at the initial phase, in which for each worker returning laden to the nest, 3 to 4 workers returned unladen (Fig. 1B). The ratio significantly decreased over time, with the lowest ratios at the end of the established phase, at which most workers returned laden to the nest (t-paired test, t_11_ = 17.98, p<0.001, Log_10_ transformed data).

**Figure 2:**
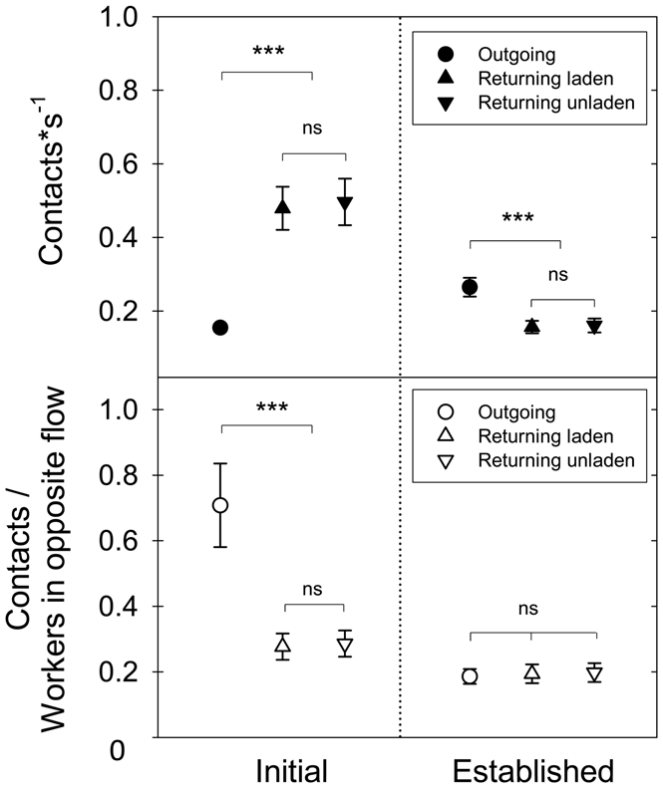
Interactions between outgoing and returning workers on the foraging trail. **Top**) Rate of head-on-contacts (mean±SD) with workers coming from the opposite direction for single outgoing, returning laden and unladen workers during both the initial and established foraging phases (Initial: F_2,33_ = 28.03, p<0.001; Established: F_2,33_ = 8.23, p<0.01; asterisks indicate a difference at p<0.01 after a Tukey post-hoc test, Log_10_ transformed data). **Bottom**) Number of head-on-contacts divided by the number of workers in the opposite flow, for both foraging phases (Initial: F_2,33_ = 9.82, p<0.001; Established: F_2,33_ = 0.009, p = 0.91, NS; asterisks indicate a difference at p<0.01 after a Tukey post-hoc test, Log_10_ transformed data).

Figure 2 summarizes the head-on encounter rates for outgoing, returning unladen and laden workers, at both foraging phases, with an average ambient temperature of 15.55°C (±SD = 0.93, N = 12) and 26.81°C (±SD = 1.28, N = 12), respectively. At the initial one, the recorded contact rates for returning workers, laden and unladen, were higher than for outgoing workers (in average, each returning worker experienced one contact every two seconds). The inverse situation was observed at the established phase (Fig. 2, top; statistics at the figure caption). Since the probability of contacting a worker directly depends on the number of workers coming from the opposite direction, and this is dependent of the foraging phase (see Fig. 1), comparative values were obtained by dividing the contact rates (contacts*s^−1^, Fig. 2, top) by the opposite traffic flow (number of workers*s^−1^, Fig. 1a). After this standardization, outgoing workers at the initial phase were significantly more likely to contact a worker coming from the opposite direction than returning workers, both laden and unladen. The observed ratio, with a value of approximately 0.7, means that 70% of the returning workers experienced a contact with an outgoing worker. This difference between outgoing and returning workers vanished at the established phase (Fig. 2, bottom; statistics at the figure caption).

Regarding their walking speed, outgoing workers moved at the initial phase at 19.73 mm*s^−1^ (±SD = 3.76, N = 36), and at the established phase at 33.02 mm*s^−1^ (±SD = 5.92, N = 36). Walking speed of returning workers was much lower at the initial phase, and similar for unladen (15.03 mm*s^−1^ ±SD = 4.32, N = 36) and laden workers (14.81 mm*s^−1^ ±SD = 4.51, N = 36; t-test, t_70_ = 0.21, p = 0.83, NS). At the established phase, walking speed was higher than at the initial phase, averaging 32.21 mm*s^−1^ (±SD = 6.93, N = 36) for unladen and 31.52 mm*s^−1^ (±SD = 7.30, N = 36) for laden ants (t-test, t_70_ = 0.41, p = 0.68, NS), being both similar to that of outgoing workers (One-way ANOVA, F_2,105_ = 0.45, p = 0.64, NS).

### Fragment-size determination, walking speed and carrying performance at different temperatures

**Figure 3:**
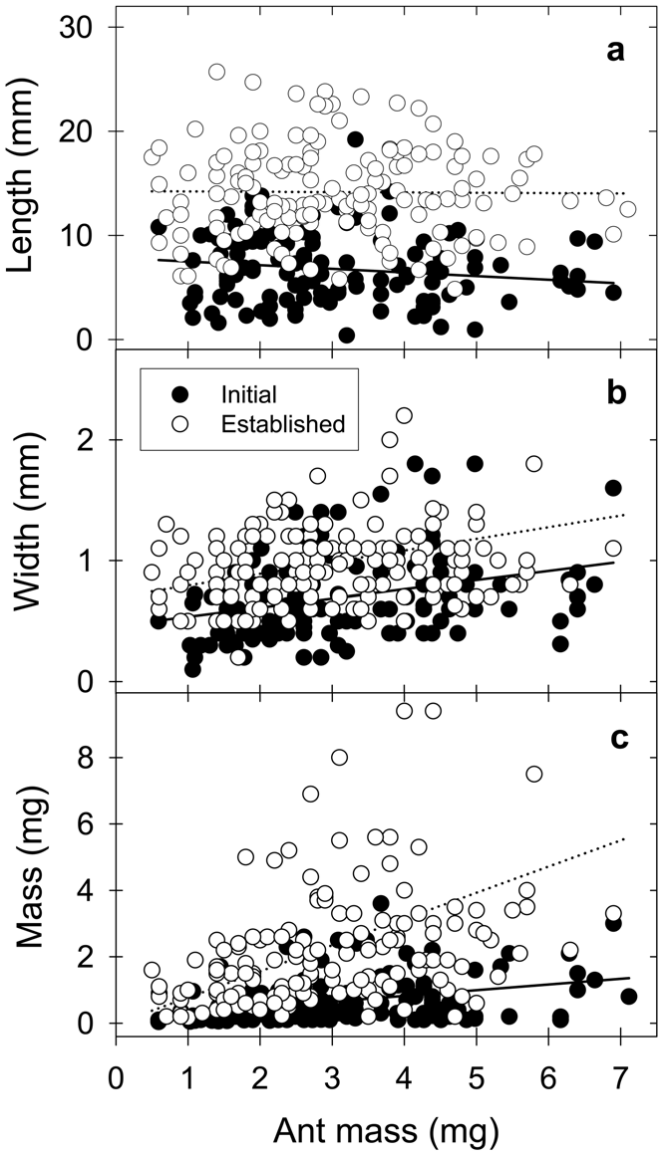
Load-size determination by foraging workers in the field. Length (**A**), width (**B**) and mass (**C**) of the grass fragments cut by different-sized foragers in the field, at both the initial and established foraging phases.

*A. heyeri* workers cut fragments of different size depending on the foraging phase (Fig. 3). The length of the fragment cut was independent of the ant size, but fragments were shorter at the initial foraging phase (Fig. 3A, ANCOVA, F_1,268_ = 232.96, p<0.001). In addition, the harvested fragments were narrower (Fig. 3B, ANCOVA, F_1,268_ = 47.81, p<0.001) and lighter at the initial foraging phase (Fig. 3C, ANCOVA, F_1,296_ = 161.38, p<0.001, Log_10_ transformed data).

Tissue area density of the fragments cut during the initial phase (median: 0.085 mg*mm^−2^, 25–75%: 0.05–0.17 mg*mm^−2^) was significantly lower than that of the fragments cut at the established phase (median: 0.13 mg*mm^−2^, 25–75%: 0.09–0.19 mg*mm^−2^, ANCOVA, F_1,268_ = 8.63, p<0.01, Log_10_ transformed data), indicating that workers selected tender grasses at the onset of foraging. To rule out the possibility that the observed differences in fragment-size determination between the two foraging phases were simply the result of workers harvesting the tips of the grass blades at the beginning of their daily foraging activity, which are likely less dense than the central and basal parts of the grass blades, the proportion of grass tips occurring in the sample of all laden workers collected was evaluated. Grass tips represented 53.2% of the loads collected at the initial phase, and 50.2% of those collected at the established phase, being these figures statistically similar (Chi-square = 0.093, p = 0.76).

**Figure 4:**
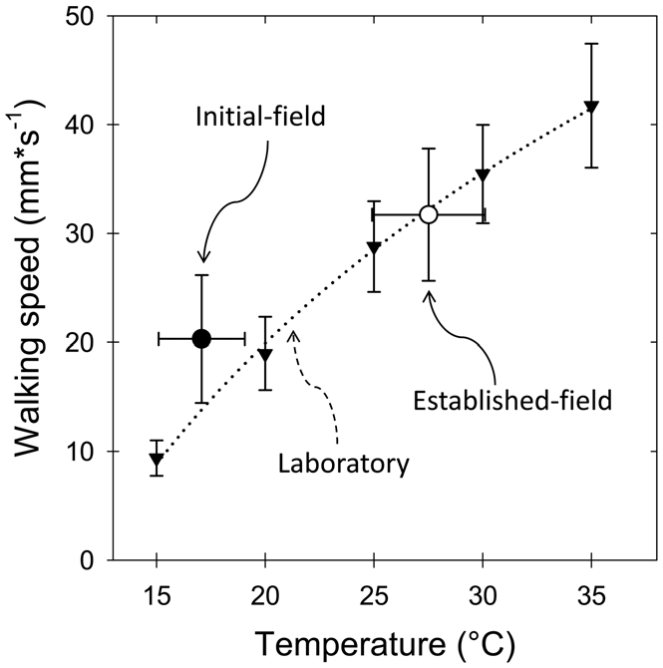
Average walking speed (±SD) of laden workers as a function of temperature, measured in the laboratory (*dotted line*: walking speed = −95.56+88.75*(log_10_T), R^2^ = 0.89). The black and white circles represent the average walking speeds (±SD) measured in the field at the indicated mean (±SD) environmental temperatures, for both the initial and the established foraging phases.

Environmental temperature markedly varied during the measurements at the two different foraging phases. Usually, the initial foraging phase started at values above 15°C (mean temperature 17.08°C ±SD = 1.98, N = 150), whereas the phase of established foraging took place at a mean temperature of 27.51°C (±SD = 2.6, N = 150). While at the initial phase the walking speed averaged 20.31 mm*s^−1^ (±SD = 5.85), at the established phase it averaged 31.71 mm*s^−1^ (±SD = 6.09). Figure 4 shows the logarithmic relationship between walking speed of laden workers and temperature over a wide range, from 15° to 35°C, as obtained under controlled laboratory conditions (Fig. 4, dotted line, black triangles). When the walking speeds measured in the field at the two foraging phases are compared to the corresponding values as measured in the laboratory, laden workers were observed to walk in average 52% faster than expected for this temperature at the initial phase, yet not at the established phase (Fig. 4, white and black circles; expected walking speed based on logarithmic fit at 17.08°C = 13.82 mm*s^−1^; expected at 27.51°C = 32.19 mm*s^−1^).

To allow comparisons of transport rates at different temperatures, field data on walking speed were transformed at 15°C using the relationship between walking speed and temperature obtained for laden workers in the laboratory (Fig. 4). Due to the observed logarithmic relationship, the fractional increase in walking speed for a temperature increase of 10°C, i.e., the Q_10_-value, was dependent of the temperature range considered. The fractional increase in walking speed for a temperature increase of 10°C is described by the equation: Q_10_ = 10*exp[10*(logV_t2_−log V_t1_)/(T_2_−T_1_)]. The Q_10_-values decreased with increasing temperatures, being Q_10_ = 4.1 for the range 15°–20°C, 2.3 for the range 20°–25°C, 1.5 for the range 25°–30°C, and 1.4 for the range 30°–35°C.

**Figure 5:**
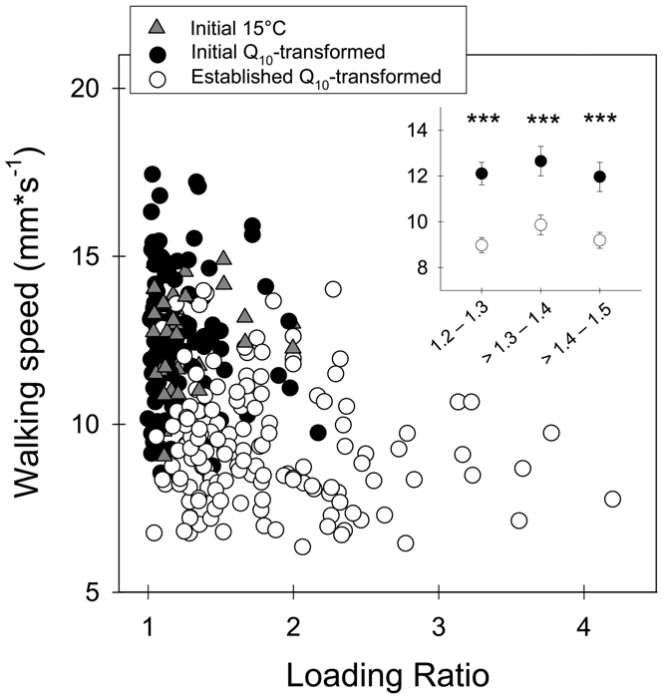
Walking speed of laden workers as a function of the Loading Ratio (LR = [Load mass+Ant mass]/Ant mass), for both the initial and established foraging phases. Speeds measured at temperatures other than 15°C, during both foraging phases, were transformed to comparable values at 15°C using the Q_10_-values obtained from the speed measurements in the laboratory, as shown in Figure 4 (further details in text). Walking speed of workers walking at 15°C (±0.1°C) during the initial foraging phase remained untransformed (gray triangles). **Inset**: Detailed view of the mean walking speeds for different ranges of loading ratios: 1.2–1.3; >1.3–1.4; >1.4–1.5. Asterisks denote statistical differences between the initial and established phase at p<0.001 (for the loading ratios values 1.2–1.3: t_32_ = 5.41; >1.3–1.4: t_31_ = 3.70; >1.4–1.5: t_31_ = 4.17).

In addition to temperature, the size of the load carried is known to markedly influence walking speed of laden workers. To rule out the possibility that the observed higher walking speeds at the initial phase (Fig. 4) resulted from the smaller loads carried as compared to the established phase (Fig. 3), load mass was standardized regarding the size of the ant that carry it, as follows: Loading Ratio = [Load mass+Ant mass]/Ant mass [28]. Figure 5 shows the relationship between walking speed and loading ratio for workers at the two foraging phases. Data collected at 15°C during the initial phase are presented as obtained; data collected at other temperatures were transformed to 15°C using the obtained Q_10_-values (see above), to allow comparisons at the same temperature. The average loading ratio was significantly lower at the initial phase (F-test, F_1,297_ = 167.94, p<0.001, Log_10_ transformed data), and walking speed (WS) negatively depended on the loading ratio (LR): WS = 13.39–1.64*LR (R^2^ = 0.14, p<0.001). As a consequence, harvesting small grass fragments at the initial phase allowed workers to return faster to the nest, as compared to the established phase. Furthermore, if workers carrying similar loads relative to their body masses are compared, i.e., workers showing similar loading ratios (Fig. 5, inset), results indicated that they returned to the nest at the initial phase faster than at the established phase, irrespective of their loads (statistics at the figure caption).

**Figure 6:**
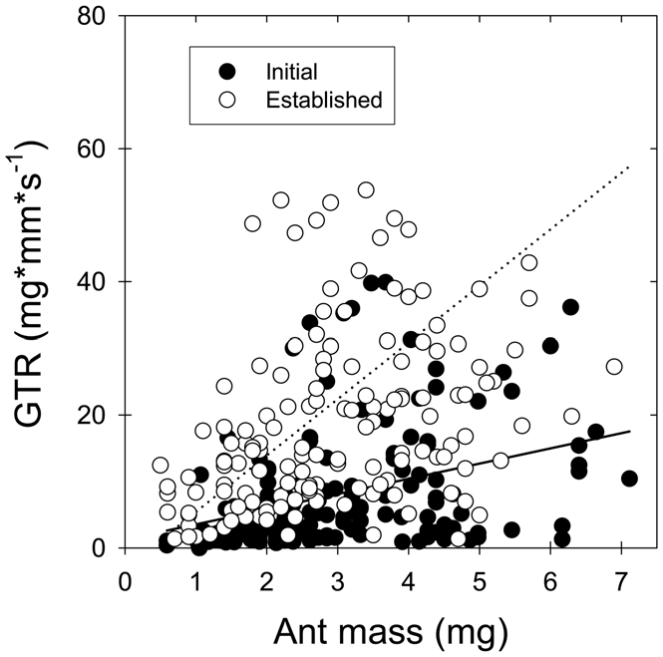
Gross transport rate of plant tissue (expressed as mass carried per mm and per second) as a function of ant body mass, for both the initial and established foraging phases. Data were Q_10_-transformed at 15°C (further details in text).

Data of temperature-corrected walking speed (at 15°C) were used to calculate the gross transport rates of vegetable tissue, a measure of individual performance, for laden workers at both the initial and the established foraging phases. For the two foraging phases, there was a positive relationship between gross transport rate and ant body mass (Fig. 6). The foraging phase had a very significant effect on the relationship between ant body mass and gross transport rate (ANCOVA, F_1,295_ = 133.68, p<0.001). Workers showed an average gross transport rate of 8.26 mg*mm*s^−1^ (±SD = 1.42, N = 148) at the initial foraging phase, lower than later at the established foraging phase, with an average of 22.61 mg*mm*s^−1^ (±SD = 26.16, N = 150).

### Cutting and lift-up times depending on the foraging phase

In the laboratory, the time needed to cut a millimeter of *Festuca* grass tissue, with a density of 0.11 mg*mm^−2^, averaged 77.12 s (±SD = 35.69, N = 19) at a temperature similar to the average value of the initial phase (15°C), and 25.27 s (±SD = 8.82, N = 19) at a temperature of 25°C, as in the established phase, being these values significantly different (t-test, t_36_ = 6.21, p<0.001, Log_10_ transformed data).

The time needed to lift-up a paper fragment similar in size to those cut in the initial phase (Fig. 3, 7 mm length, 1 mm width, 0.9 mg) averaged 41.91 s (±SD = 18.60, N = 30) at 15°C, the mean temperature at the initial foraging phase in the field. For a paper fragment as harvested in the established phase (Fig. 3, 14 mm length, 1 mm width, 2.2 mg), lift-up times were in average much longer, 83.62 s (±SD = 57.58, N = 30; t-test, t_58_ = 4.49, p<0.001, Log_10_ transformed data). At 25°C, the time needed to lift-up a paper fragment similar in size to that cut at the established phase averaged 37.02 s (±SD = 23.37, N = 30), while for a fragment similar in size to that cut at the initial phase, the lift-up time was significantly shorter, averaging 15.21 s (±SD = 6.57, N = 30; t-test, t_58_ = 5.82, p<0.001, Log_10_ transformed data).

### Cutting, loading and carriage: overall returning times by laden and unladen workers

Taken into account the cutting times, handling times and walking speeds, the overall returning time at the *initial foraging phase* can be calculated for *i)* unladen workers, which do not spend any cutting or handling times, *ii)* workers carrying loads as they select at the initial foraging phase, and *iii)* workers that would not decide to select smaller loads as expected, but harvest larger loads as it occurs in the *established foraging phase*. Calculations are based on a returning trip along 20 meters, which is the average *A. heyeri*'s trail length (personal observations). Since the average walking speed of unladen and laden workers did not differ, the returning time of *unladen* workers, based on the speed of laden workers (Fig. 5, black circles), would average 26 min 40 s. For *laden* workers carrying small loads as observed in the initial phase at 15°C, both the time needed to cut a grass of 0.68 mm width (Fig. 3B), and to lift-up the resulting fragment of 7 mm length (Fig. 3A) should be added, resulting in a returning time of 28 min 35 s. For *laden* workers that would not decide to cut a small fragment at 15°C, but a larger one as in the established phase, both the time needed to cut a grass of 0.98 mm width (Fig. 3B), and to lift-up the resulting fragment of 14 mm length (Fig. 3A) have to be added, as well as the additional travel time resulting from the carriage of a larger load at lower speed (Fig. 5, white circles). The returning time for those workers would average 38 min 22 s. Taken together, *A. heyeri* workers saved between 30% and 26% of their time by returning unladen or with smaller loads as observed at the initial foraging phase, respectively.

## Discussion

### Foraging phases, contact rates, and information transfer

Leaf-cutting ants initiate their daily foraging activity by leaving the nest in large numbers without the need of recruitment [16], as also observed in the investigated field colonies (Fig. 1). Foragers search for suitable resources and, upon discovery, decide whether a given resource is worth communicating to others. The acceptance of a plant is mainly based on chemical and physical features of its leaves [29], and also on the workers' foraging experience [30]. If the source is suitable, workers return to the nest (laden or unladen) laying a chemical trail, and interact with nestmates both on the trail and inside the nest [5], [10], [14]. Our field measurements showed that at the beginning of their daily foraging activity, *A. heyeri* workers had a greater tendency to return to the nest unladen. The proportion of unladen workers diminished over time, and attained the lowest values at the time when the traffic flows to and from the nest reached roughly similar values.

Why a large proportion of workers, at the initial foraging phase, returned to the nest unladen? It is known that over an entire foraging process, this proportion may be high, varying between 13% and 75% [21], [31]–[33]. While a number of these unladen workers can be involved in trail-clearing [34] or transport of plant sap [35], others are engaged in the reinforcement of the chemical trail or in a combination of food transport and recruitment communication [14], [36]. The observed interactions between outgoing and returning workers, and published results [9], [18], provide indirect support to the idea that the decision to return unladen to the nest, with the concomitant time savings, represents a response to the high needs for information at the beginning of a daily foraging process. Under the field conditions of our study, it was unfeasible to quantify the intensity of recruitment communication, so that direct evidence for information transfer is lacking. Returning workers were not necessary to stimulate workers to leave the nest, which already occurred in large numbers on the trail at the time the first workers returned to the nest (Fig. 1) [16]. We suggest that returning workers, through their interactions with outgoing workers on the trail and the expected trail-marking, are responsible for the initial establishment of a foraging process. Outgoing workers, after contacting returning workers and following the chemical trail, are expected to arrive shortly thereafter at the discovered source, and make foraging decisions (flexible fragment-size determination, trail-laying, further contacts) that amplify and help maintain the foraging process already initiated.

Head-on encounters between workers moving in opposite directions were very frequent, dependent on the foraging phase, and different for outgoing and returning workers, even after standardization to the traffic flow. This clearly indicates that such contacts did not simply represent collisions in a probabilistic sense. Interestingly, outgoing workers at the initial phase were significantly more likely to contact a worker coming from the opposite direction than returning workers, both laden and unladen. Counts indicated that each outgoing worker contacted in average 70% of the returning workers at the initial phase, which suggest an active search for contacts and/or information, and only 20% at the established phase. Such an active search was described for *Atta cephalotes* as early as 1929 by Lutz, who observed that “frequently, a returning laden forager is stopped momentarily by an outgoing nestmate which is apparently interested in what is being carried” [16]. Returning foragers, on the contrary, showed similar contact rates at the two foraging phases. It appears that they do not actively search for contacts, but just return as fast as possible to the nest, likely laying a chemical trail [14]. Outgoing *Atta cephalotes* workers are also known to experience higher encounter rates than returning ants [37], and it has been speculated that they may actively seek encounters with returning ants for information acquisition. The present results go beyond those findings by showing that the contact rates experienced by outgoing workers depended on the time since the onset of foraging, being initially very high, and dropping drastically when a foraging column was already established. It can be argued that the active search for interactions in outgoing workers represents an indirect measure of the workers' information needs, which is strongly phase-dependent. Even though we were unable to establish whether such a contact indeed involved information transfer or not, it is known that foraging decisions of outgoing workers, and their probability to find a recently-discovered food source, are influenced by the interactions with returning nestmates and the odour of the fragment they carry [10], [38]–[41]. In fact, there is a large body of evidence, particularly for harvester ants, emphasizing the role of ant encounters for the regulation of foraging activity [11], [42]–[45]. Even the mere fact of an encounter may provide information, such as the magnitude of the colony's foraging activity, and therefore influence the probability of food collection in ants [46]–[48].

### Fragment-size determination: individual vs. social demands

*A. heyeri* foragers cut grass fragments of different size depending on the time since the onset of their foraging activity. Fragments were shorter, narrower and lighter at the initial foraging phase, and workers selected in addition tender grass blades for harvesting, i.e., grasses with lower area density. Considering that load size has a large influence on maneuverability and speed of transport and, therefore, on the rate of plant tissue delivery [20], [24], [25], [49], foragers should *a priori* be expected to carry loads that maximize their individual delivery rate, i.e., the amount of plant tissue carried to the nest per unit of foraging time.

Do *A. heyeri* workers perform different as individual carriers, depending on the foraging phase? The observed differences in environmental temperature during both foraging phases, which markedly influence locomotion speed of ectothermic animals like ants [50], preclude a direct comparison of individual transport rates between phases. By converting the absolute velocities to standardized values at 15°C (using Q_10_-values for walking speed measured in the laboratory), individual transport rates at both the initial and established foraging phases could be properly compared. Our results showed that even though workers at the initial foraging phase walked at a significant faster pace than expected for this temperature (Fig. 4 and Fig. 5), their individual transport rate was markedly low, averaging 37% of the rate observed at the established foraging phase (Fig. 6), as known for the leaf-cutting ant *Atta cephalotes* under controlled laboratory conditions [18]. Interestingly, laden workers walked at a fast pace despite their rate of encounters with outgoing workers, which moved in large numbers at the beginning, suggesting that returning workers experienced no significant delays on their way caused by these encounters.

### A worker's foraging cycle: when is time worth saving?

The observed differences in fragment-size determination at the two different foraging phases have a number of consequences on both individual performance and colony-wide patterns. At the individual level, cutting length and therefore cutting time was shorter at the initial foraging phase, because of the selection of narrow grasses. The time needed to lift-up the selected fragments was also shorter than the time needed for a larger fragment, as selected at the established phase. And, in addition, workers were able to walk faster because of the transport of lighter (shorter) grass fragments. Plasticity in workers' cutting rules at the initial phase, compared to the alternative of cutting larger fragments as in the established phase, led therefore to a reduction of time costs of around 30%. At the colony level, such time savings may allow a higher frequency of individual roundtrips during a daily foraging process, with the resulting increase in the probability of interacting with outgoing workers for information transfer.

As mentioned above, the foraging phase did not only influence cutting behaviour, but walking speed of laden foragers (Fig. 4). By comparing the walking speeds at the two foraging phases with the speeds measured in the laboratory at the same temperatures, workers were observed to walk faster than expected at the initial phase, yet not at the established phase. Moreover, when controlling for differences in the fragment-size carried, workers still walked faster at the initial phase than at the established phase (Fig. 5, inset). It follows that beyond the expected physiological effects of temperature on ant locomotion [50], [51], an additional drive motivates workers at the initial foraging phase to increase their walking speed, as described for a related species [5], [9]. It is an open question how outgoing workers identify the actual foraging phase, so as to adjust their behaviour accordingly. Workers may acquire information via the rate of contacts they experience, the probability of encountering laden workers on their way to the food source, or respond to potential differences in the intensity of pheromone marking on the trail.

Why do laden workers walk faster at the initial foraging phase, but carry less material than later at the established phase? The adaptive value of this response, with the associated time savings, might be related to the high information needs at the time of a food discovery, thus allowing both a faster establishment and the maintenance of a foraging process. We argue that at the initial phase of trail development, saving time would be of great importance to monopolize a food source as soon as possible. In fact, the extreme strategy of time-saving at the source for information transfer is that described for the leaf-cutting ant *Atta cephalotes*: scout workers, upon discovery of a newly-food source, cut no fragments at all but return quickly to the nest laying chemical trails, and only start cutting leaves when a foraging column has been established [14]. Whether information needs also influences load-size determination under routine foraging conditions on well-established trails, is unclear [52].

In conclusion, our results support the hypothesis that at the initial foraging phase, workers compromise their individual transport rates of material in order to return early to the colony, as predicted by the information-transfer hypothesis [2], [9], [22]. The selection of partial loads at the beginning of foraging, as well as the decision to return to the nest unladen, appear to be driven by the need of a rapid information transfer at the initial phase of the monopolization of resources [18], beneficial in an environment where the foraging time windows are constrained by environmental variables [33], and neighbouring colonies may compete for resources [53].
